# British Columbia community pharmacy during COVID-19: Describing the patient experience via Google reviews

**DOI:** 10.1177/17151635241281749

**Published:** 2024-10-30

**Authors:** Priya Bains, Fong Chan, James P. McCormack, Aaron Sha

**Affiliations:** Faculty of Pharmaceutical Sciences, University of British Columbia, Vancouver, BC; Faculty of Pharmaceutical Sciences, University of British Columbia, Vancouver, BC; Faculty of Pharmaceutical Sciences, University of British Columbia, Vancouver, BC; Faculty of Pharmaceutical Sciences, University of British Columbia, Vancouver, BC

## Introduction

Since March 2020, the COVID-19 pandemic has significantly affected the Canadian health care system, and its repercussions continue to be felt today.^1^ More than 500,000 surgeries were cancelled or delayed over the first 16 months of the pandemic, virtual care by physicians through phone or online accounted for 27% to 57% of physician services in 5 provinces, and hospitals continued to shift resources to serve intensive care units.^1^ Pharmacists and community pharmacies were also affected on several fronts during the pandemic and faced unprecedented challenges in providing care in the best interest of their patients and staff.^2^

Since the first wave of COVID-19 in British Columbia (BC), the Ministry of Health and the College of Pharmacists of BC (CPBC) have announced several emergency responses to the pandemic. In March 2020, BC community pharmacies were tasked to provide emergency supplies and prescription refills to avoid nonessential physician services.^3^ This led to increased demand on drug supplies and subsequent drug shortages, leading to some pharmacies rationing medications, which led to patient dissatisfaction.^4^ In November 2020, the BC Minister of Health announced mandatory masking in all indoor public spaces, leading to a huge demand for masks and thus an increased pharmacy workload, given that pharmacists are the primary provider of personal protective equipment.^5^ Additionally, as vaccines and COVID-19 testing kits became more widely available, community pharmacies were also tasked with vaccinations and test distribution, leading to increased time demand on pharmacy professionals. All the above factors and others have changed the public’s expectation of community pharmacies.

Online reviews have been a valuable tool in assessing patient satisfaction in health care. Although the value of online reviews can be controversial, as they may lack transparency, may be monetarily motivated or are sometimes left in error, there is no denying technology has become intertwined with most retail settings and is one of the most convenient ways for patients to leave direct feedback.^6^ Google reviews have been studied in the health care setting. They have been used to assess the quality of care in nursing homes and evaluate patient satisfaction of dermatology providers as well as emergency departments.^6-8^ Yelp reviews have been analyzed in the pharmacy setting and key words such as “wait time” have been associated with lower-star ratings.^9^ However, the full length of the reviews was not analyzed to provide the full context of these lower ratings.^9^

To date, there have been no published studies using Google reviews to evaluate patient care in community pharmacies. In our exploratory study, we analyzed Google reviews collected in the rapidly changing pharmacy landscape before and after the COVID-19 pandemic in BC to better understand opportunities and challenges for community pharmacy teams.

## Methods

### Study population

People who received community pharmacy services in BC who have left Google reviews regarding their pharmacy experience were included in this study. The list of licensed community pharmacies from the College of Pharmacists of BC, collected in November 2022, was used to determine Google review pages. All BC pharmacies were included except those that did not provide direct community patient care (e.g., online delivery only, central fill). Pharmacies with no reviews or no relevant reviews were also excluded. Relevant reviews contained reviewer comments that directly mentioned interaction with pharmacy or pharmacy staff (e.g., received medication/prescription, talked to pharmacy staff member, received vaccines). Irrelevant reviews had no direct interaction with pharmacy or pharmacy staff (e.g., eggs were not on sale, cosmetician was nice in providing makeup). For post-COVID results, reviews were collected from March 12, 2020, the first day of COVID-19 restrictions, to October 14, 2022, the date when the expanded scope of pharmacists became active. For comparison, pre-COVID reviews were collected from August 8, 2017 to March 11, 2020, which is a similar time span used for the post-COVID data.

### Data collection

Google reviews data with date ranges were scraped from Google servers using Outscraper, a cloud-based platform that extracts public data from the Internet, including Google reviews. Outscraper can be used to generate data sets of reviewer comments and provide exact dates, location and rating. Two reviewers independently sorted all the reviews as relevant vs irrelevant based on the above criteria, and consensus was established before including reviews in the study. In the case where reviewers were unclear whom the comment was given to, the review was considered irrelevant. Due to the complex and diverse nature of Google review comments, good reviews were defined as reviews that contained a 4- to 5-star rating. When a review gave a 3-star rating, comments were further classified into good, neutral or bad based on content and sorted into good, neutral or bad reviews. Two independent reviewers analyzed all comments and came to a consensus before including reviews in the above categories. Bad reviews were reviews that had 1- to 2-star ratings. Comments in reviews were further categorized into pharmacist professionalism, cost, wait time, knowledge, scope of practice (e.g., pharmacist’s willingness to provide emergency supply) or other (e.g., accessibility and organization of flu clinics). For post-COVID reviews, comments were further subcategorized into prevention measures, vaccine-related, testing-related or other. Word clouds of positive and negative comments were also created using TagCrowd software to identify descriptive key words associated with patient experience. For word cloud generation, the top 30 words most associated with positive and negative reviews are presented. Common words, including *pharmacy*, *pharmacist*, *prescription*, *service* and *staff*, were excluded.

## Results

### Pharmacies and Google reviews

**Figure 1:**
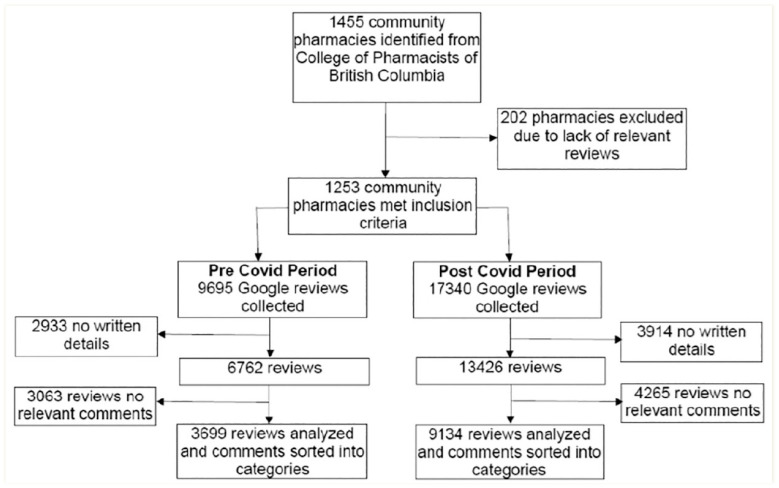
Pharmacy identification and Google review selection

Of the 1455 community pharmacies identified from the CPBC website, 1253 pharmacies had relevant Google reviews within the study periods. Reviews were analyzed for relevance to pharmacy practice, and 3699 reviews met inclusion criteria in the pre-COVID period and 9134 in the post-COVID period (Figure 1).

### Review analysis

Of the pre-COVID reviews, 75% were perceived to be positive, 24% were negative and 1% were neutral. In comparison in the post-COVID reviews, 69% were positive, 30% were negative and 0.3% were neutral (Table 1). Review comments were further grouped into categories (Table 2). Comments were not mutually exclusive, meaning 1 review could apply to multiple categories. Professionalism was mentioned most frequently in both positive and negative reviews during both time periods. Knowledge of the pharmacists was the second most commented topic for the positive reviews (24% pre-COVID and 15% post-COVID), followed by wait time and cost. In terms of negative reviews, the second most commented topic was regarding wait time, followed by knowledge and cost. Scope of practice was rarely mentioned in both pre- and post-COVID periods, accounting for <2% of comments in all reviews.

**Table 1:** Google review ratings for included pharmacies

	Pre-COVID (*n* = 3699), %	Post-COVID (*n* = 9134), %
Positive review	75	69
Negative review	24	30
Neutral review	0.7	0.4

**Table 2:** Google review comments categorized

	Pre-COVID-positive (*n* = 4227), %	Pre-COVID-negative (*n* = 1187), %	Post-COVID-positive (*n* = 8718), %	Post-COVID-negative (*n* = 3272), %
Cost	5	7	3	5
Professionalism	59	47	60	44
Knowledge	24	20	15	9
Scope of practice	0.3	2	0.4	2
Wait time	12	23	12	26
COVID-19 related			7	10
Other	0.4	2	3	5

### Post-COVID reviews

Reviews containing comments relating to COVID topics were mostly positive (75%) (Table 3). For positive reviews, most of the comments were about availability/delivery of the vaccine (86%), followed by proper prevention measures (5%) and provision of COVID tests (5%). Negative reviewers commented mostly on vaccine-related issues (31%), followed by COVID testing availability (24%) and prevention measures (12%).

**Table 3:** COVID comments subgroup analysis

	Positive (*n* = 620), %	Negative (*n* = 211), %
COVID test	5	24
Prevention measures	5	12
Vaccine	86	31
Other	3	11

### Word cloud

**Figure 2:**
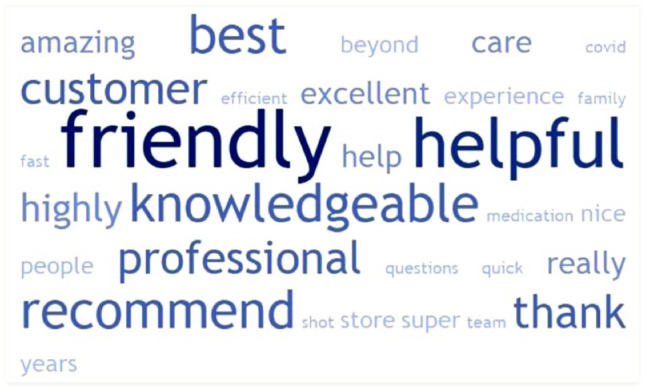
Word cloud of top 30 words from positive Google reviews

**Figure 3:**
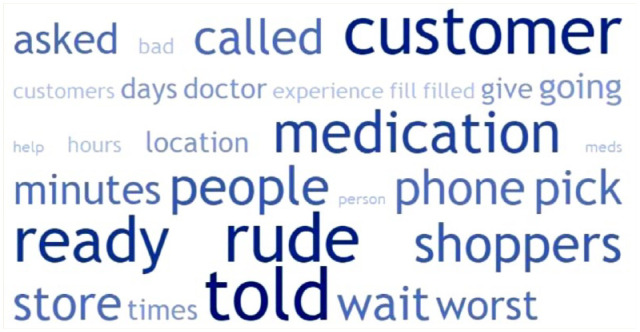
Word cloud of top 30 words from negative Google reviews

The top 30 descriptive key words found in post-COVID reviews are presented in Figures 2 and 3. For positive reviews, the words related to staff professionalism, such as *friendly* and *helpful*, were most prominent. Similarly, in the negative reviews, *rude* and *told* appeared more frequently. The word shoppers, which is one of the biggest pharmacy chains in Canada [Shoppers Drug Mart], was also popular among negative reviews as people would blame the chain for the negative review. This differs from positive reviews where people would mention the specific pharmacist by name when they had a positive experience.

## Discussion

Online reviews are increasingly important to how consumers choose places to shop, eat and receive medical and pharmaceutical care. Our data have shown that 75% of patients who left Google reviews of community pharmacies in BC had a positive experience during the pre-COVID dates (August 8, 2017 to March 11, 2020) compared to 69% during the post-COVID dates (March 12, 2020 to October 14, 2022). The number of Google reviews for pharmacies increased over time, possibly due to increased use of pharmacy services during lockdown periods and popularization of online reviews. Professionalism was the most frequent discussion in both positive and negative reviews, accounting for almost 60% of all positive reviews and half of all negative reviews. Knowledge, wait time and costs were less common factors in determining patient experience. Our results echo those of online review analyses of other health professionals such as dermatologists and surgeons, which also found the physician’s bedside manner was the principal factor over wait time and costs.^6^ Of note, reviewers commented infrequently on the pharmacist’s scope of practice, accounting for only 2% of all comments. This may be due to the lack of public awareness of the pharmacist’s increasing scope of practice. With common ailment prescribing in BC starting in June 2023, comments regarding pharmacists as primary care providers, an example of the increasing scope of practice, may become more commonplace.

Our data can provide strategies to enhance patient satisfaction by focusing on factors that influence patients’ sentiments and trust. Specifically, these data can guide pharmacy practice by pinpointing areas where our profession should prioritize, such as improving professionalism vs wait time. For example, in both before and after COVID-19 periods, professionalism was the most frequent discussion, meaning reviewers found professionalism to be the most noticeable aspect of their pharmacy experience. On the opposite side, less than 2% of all comments included pharmacist scope of practice, meaning reviewers did not use this service or find the service noteworthy. Our research can guide pharmacy practice to improve professionalism training provided to pharmacy staff and focus on the opportunity to advertise and better utilize our expanded scope.

In our post-COVID review subanalysis, we found 75% of comments were positive regarding COVID-19 prevention measures, vaccine and testing. The data showed that 86% of positive comments were regarding vaccine administration and availability, as pharmacists have become the top provider of vaccinations in the province. Negative reviewers commented on vaccine-related issues, testing availability and prevention measures. This research highlighted the positive impact community pharmacies made in providing supplies and vaccinations and that reviewers had a negative experience when they were unable to access these services at their local pharmacy, which was beyond the control of the pharmacists.

There are several limitations in this study. First, there is the inability to confirm reviewer identity and veracity. Positive Google reviews can be purchased through websites, and negative reviews can be removed by appeal, which may have skewed our results. However, by including only reviews that had relevant written comments, we may have removed some purchased 5-star rating reviews. Another limitation is that Google reviews have high responder bias, as people who have particularly good or bad experiences are more likely to write a review. This study can only speak for the people who left a review, which can give us insight into what factors within the scope of pharmaceutical care patients value as important, but these data were not representative of the entire population.

## Conclusion

Our study is the first to analyze Google reviews in their entirety to provide insight into what patients comment on when receiving care in community pharmacies in BC. Our data can be used to provide strategies to improve patient satisfaction by focusing on factors that influence patients’ sentiments and trust. These data can direct pharmacy practice by highlighting which areas our profession should focus on, like improving professionalism in the workplace vs cost of items. Our study has also shown the impact of community pharmacies in providing supplies and vaccinations during a pandemic and the challenges patients face in obtaining COVID-related care. The scope of pharmacy practice has been evolving in Canada, most recently the expanded scope of practice for pharmacists in both BC and Ontario, which will warrant further online review analysis in the future to better understand awareness and attitudes towards enhanced pharmacy services.
